# Structural Monitoring of Underground Structures in Multi-Layer Media by Dynamic Methods

**DOI:** 10.3390/s20185241

**Published:** 2020-09-14

**Authors:** Alexandr Lyapin, Alexey Beskopylny, Besarion Meskhi

**Affiliations:** 1 Department of Information Systems in Construction, Faculty of IT-Systems and Technologies, Don State Technical University, Gagarin, 1, Rostov-on-Don 344000, Russia; lyapin.rnd@yandex.ru; 2 Department of Transport Systems, Faculty of Roads and Transport Systems, Don State Technical University, Gagarin, 1, Rostov-on-Don 344000, Russia; 3 Department of Life Safety and Environmental Protection, Faculty of Life Safety and Environmental Engineering, Don State Technical University, Gagarin, 1, Rostov-on-Don 344000, Russia; reception@donstu.ru

**Keywords:** layered structures, dynamic, wave propagation, impact, underground structure, stress-strain state

## Abstract

The actual problem of structural monitoring and modeling of dynamic response from buried building is considered in the framework of arbitrary dynamic load. The results can be used for designing underground transport constructions, crossings, buried reservoirs and foundations. In existing methods, the system of sensors that register the response to a dynamic action does not allow for effective interpretation of the signal without understanding the dynamic features and resonance phenomena. The analytical and numerical solution of the problem of the dynamics of a buried object in a layered medium is considered. A multilayer half-space is a set of rigidly interconnected layers characterized by elastic properties. At a distance, an arbitrary dynamic load acts on the half-space, which causes oscillations in the embedded structure, and the sensor system registers the response. The problem of assessing the dynamic stress-strain state (DSSS) is solved using Fourier transforms with the principle of limiting absorption. As an example, the behavior of an embedded massive structure of an underground pedestrian crossing under the influence of a dynamic surface source on a multilayer medium is considered, as well as instrumental support of the sensor system. The solution in the form of stress, strain and displacement fields is obtained and compared with the experimental data. The frequency-dependent characteristics of the system are determined and the possibility of determining the DSSS by a shock pulse is shown.

## 1. Introduction

One of the critical tasks in the industry is diagnostics of the state and dynamic structural monitoring of constructions in a multilayer media to ensure their reliability and safe operation [1]. At the same time, the features of wave propagation in layered media, multiple reflections of disturbances from the boundaries of layers make such problems very difficult to interpret. Instrumental support of dynamic tests of buried objects is inevitably associated with the solution of dynamic problems of wave propagation and assessment of the stress-strain state of structures under various influences.

Problems of the dynamics of layered media have long attracted the attention of researchers. Many concepts of strength have changed with the advent of laminates. Damascus steels used to make weapons have excellent toughness and cutting properties due to their layered structure. Bulletproof vests are made of composite laminates and revolutionized personal firearm protection.

The geophysical structure of the Earth’s surface makes it possible to formulate the problems of the dynamics of buildings and structures lying on layered media under seismic influences. The dynamic behavior of buildings and structural elements under seismic influences are considered in the works of Terenzi et al. [2], Di Lorenzo et al. [3], and Sabino et al. [4].

At the same time, it is being improved as an instrumental base for digital measurements (Li Zhu et al. [5], Xiao-Wei Ye et al. [6]), and methods of signal processing and analysis. Anaissi et al. [7] presented a new algorithm for detecting and assessing damage in bridge structures. This technique uses tensor analysis to combine data and feature extraction, and additionally uses one-class support vector machines to detect structural damage. The indirect boundary element method was proposed by Ba et al. [8] to study three-dimensional (3D) dynamic responses of a two-dimensional (2D) hill in a layered elastic half-space subject to obliquely incident waves. The proposed method is applied to construct a scattered field in closed areas.

The spatial problem (3D) of the dynamic interaction of the soil structure was investigated by Hana et al. [9] using a model of a system with several degrees of freedom on a cylindrical rigid foundation embedded in a layered fluid-saturated poroelastic half-space using the indirect boundary element method (IBEM).

An efficient method for calculating layered composite materials was proposed in [10,11] based on the contact layer model.

The problems of non-destructive testing (NDT) and monitoring of the state of structures of engineering structures, such as oil and gas pipelines, rails, aircraft components, and adhesive joints, are considered in the work of Lugovtsova [12]. Modeling and analysis of wave propagation and their interaction with damage due to dispersion and multimodal nature was carried out on the basis of the finite element method with a scaled boundary. The results obtained made it possible to analyze the propagation of waves in multilayer structures and are important for the further development of non-destructive testing of engineering structures consisting of several layers.

The dynamics of layered structures is effectively used in the analysis of the stability of landslide slopes. In the works of Zhan et al. [13], the behavior of landslide slopes under the action of dynamic loads was considered. Experimental studies by Zou et al. [14] made it possible to simulate the behavior of bridge elements with different peak accelerations. Evaluation of the behavior of earthen dams was performed by Cai et al. [15]. The design and analysis of the destruction of sand structures was carried out by Zhou [16] to study their stability under dynamic loads. Using nonlinear and machine learning methods, i.e., grouped data processing (GMDH) and multivariate adaptive regression spline (MARS), two models were applied and developed to predict slope deformation in earth dams for different types of earthquakes. An analytical solution was presented by Guo [17] based on a limit equilibrium analysis.

The influence of geological factors of the coal seam on the initiation pressure and crack propagation under dynamic impacts was studied by Cao et al. [18]. As a result of theoretical analysis, a model was created for calculating pressure on a multilayer coal seam based on the fracture criterion in the theory of maximum tensile stress.

Analysis of the dynamic response of heterogeneous layered media with the aim of developing a method of NDT and determining their mechanical and strength characteristics was carried out in [19,20]. A comprehensive diagnostic method was proposed in [21] based on the analysis of the unsteady response of a multilayer structure, on the surface of which a shock load acts. During the solution procedure, various methods of genetic algorithms are used which have shown good results. The NDT method (Bertocci et al. [22]), based on scanning acoustic microscopy, for damage detection becomes very useful for detecting defects in multilayer structures with a thickness of several microns, providing a low time investment (limitation for other NDT methods) and quantitative analysis based on measurements.

Some problems of nonstationary dynamics are solved by direct methods using numerical analysis. Kuvat et al. [23] applied methods for determining the dynamic properties of mixtures of sand and bitumen, which can be used as damping materials in the foundations of building structures. In cyclic tests, the influence of the content and properties of bitumen on the ability to absorb energy was studied.

The design of buildings and structures under dynamic loads from moving vehicles is considered in the works of Zhou et al. [24] and Ai et al. [25]. Sun et al. [26] noted that high-performance roads require a reliable tool that can calculate the structural response caused by moving vehicles. Models based on the method of spectral elements are proposed for efficiently predicting the three-dimensional dynamic response of layered systems under a moving load. The study of the dynamic characteristics of a layered poroelastic soil lying under an uneven pavement exposed to traffic loads was carried out by Lyu et al. [27]. The pavement layer is modeled as a thin Kirchhoff plate, while the underlying base and substrate are treated as a saturated two-layer poroelastic medium. A numerical verification of the proposed models allows one to accurately predict the dynamic response of layered systems caused by a moving load.

Algorithms for artificial intelligence and creation of a neural network were developed in the articles by Beskopylny et al. [28] and Lyapin [29] based on direct algorithms for solving (Lyapin et al. [30]). The data of the response of the time domain of the multilevel structure to the diagnostic shock load are used as input information. This approach provides a tool for restoring the elastic properties of layers according to the corresponding thickness coordinates.

The above analysis of the literature shows that many problems of the dynamics of multilayer media are very relevant, are of great practical importance, and are solved either numerically or experimentally. At the same time, the dynamic features of wave reflections from the boundaries of media and the resonance properties of objects can be determined with great difficulty from the numerical or experimental analysis. In addition, in the process of designing a sensor system, frequency-dependent characteristics of the environment appear, leading to nonlinear responses and complicating data analysis. Thus, the purpose of this article is to construct an analytical method that allows simulating dynamic effects in layered media, as well as obtaining fundamental solutions, identifying resonance zones and features of the dynamic response of a structure using the example of underground structures.

## 2. Analytical Approach to the Problem of the Underground Object Dynamics in a Layered Medium

**Figure 1:**
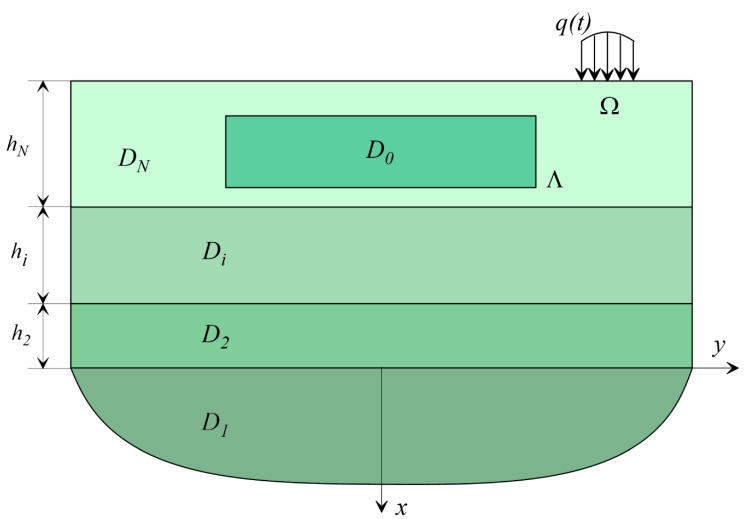
Layered half-space scheme with an arbitrary underground structure.

Let us consider the area occupied by a linear elastic (viscoelastic) medium, which is a multilayer half-space, under the surface of which an underground structure of arbitrary shape is located (Figure 1).

A multilayer half-space consists of a set of layers $D=D_{1}\cup D_{2}\cup \ldots \cup D_{N}\cup D_{0}$ where $D_{1}=\left\{x>0;\;\;y\in \left(-\infty ,+\infty \right)\right\}$ is occupied by the half-space. $D_{j}=\left\{x\in (-x_{j},-x_{j-1});\;\;y\in \left(-\infty ,+\infty \right)\right\},\;\;x_{j}=\sum_{i=2}^{j}h_{i};\;\;$ is *j*th layer (*j* = 2, …, *N*); and $D_{0}$ is the area occupied by an underground structure.

The physical properties of the medium are described by the density $\rho_{j}$ and propagation velocities of transverse and longitudinal waves $V_{Sj},V_{Pj}$.

The conditions for joining dissimilar layers are considered rigid with the requirement of continuity of the vectors of displacements and stresses when passing through the interface.

Without limiting generalization, rigid connection conditions can be changed to homogeneous frictionless contact conditions including continuity of normal displacements and absence of tangential stresses as well as Coulomb friction.

On the surface of the medium outside the structure in a limited area Ω, a system of forces q(t) oscillating with a frequency ω is set.

The wave fields for the displacements of the foundation points and the layered medium are described by integral representations (1) obtained on the basis of the dynamic reciprocity theorem

$$\begin{array}{c} u_{}^{\left(0\right)}(r_{0})=\int_{\gamma }^{}T^{*}(r_{0},r)\cdot n(r)\cdot u_{}^{\left(0\right)}(r)ds+\int_{\Lambda }^{}U^{*}(r_{0},r)\cdot \tau (r)ds+\\ +\int_{\Omega }^{}{U^{*}(r_{0},r)|}_{x=0}\cdot q(y)dy; \end{array}$$

(1) $$\begin{array}{c} u_{}^{}(r_{0})=-\int_{\gamma }^{}T^{**}(r_{0},r)\cdot n(r)\cdot u(r)ds+\int_{\Lambda }^{}U^{**}(r_{0},r)\cdot \tau (r)ds+\\ +\int_{\Omega }^{}{U^{**}(r_{0},r)|}_{x=0}\cdot q(y)dy\;. \end{array}$$

The components of the displacement vector $u_{}^{\left(0\right)}(r_{0})$ at the boundary γ of the final body (structure) and the stress vector τ(r) in the area Λ of joining the structure with the layered foundation are unknown. Functions describing stresses and displacements at a point r for a homogeneous plane with a source $r_{0}$ are selected as fundamental solutions $T^{*}(r_{0},r),\;U^{*}(r_{0},r)$ for the region $D_{0}$.

For a layered foundation, the matrices of fundamental solutions $T^{**}(r_{0},r),\;U^{**}(r_{0},r)$ are implemented using the superposition principle. This construction of a solution for a multilayer medium is based on the derivation of constitutive relations for one layer with stress vectors specified on its faces [26].

Let, in the local coordinate system for the *j*th layer $\left(x,y\right):\;x\in \left(0,h_{j}\right)\;,\;\;y\in \left(-\infty ,\infty \right)$, the amplitude functions of displacements under the action of a lumped source $r_{0}$ have the form

(2) $$U_{}^{{}^{\left(**\right)}}(r_{0},r)={\left\{U_{1}^{{}^{\left(j\right)}}(r_{0},r),U_{2}^{{}^{\left(j\right)}}(r_{0},r)\right\}}^{T}={\left\{U_{x}^{{}^{\left(j\right)}}(r_{0},r),U_{y}^{{}^{\left(j\right)}}(r_{0},r)\right\}}^{T}$$

The functions $U_{k}^{\left(j\right)}(r_{0},r)$ satisfying the equations of motion, according to the proposed method are sought in the form

(3) $$U_{k}^{{}^{\left(j\right)}}=U_{k}^{{}^{\left(j,1\right)}}+U_{k}^{{}^{\left(j,2\right)}}+U_{k}^{{}^{\left(j,3\right)}}$$

In a similar consideration of the half-plane, we assume $U_{k}^{\left(j,1\right)}\equiv 0$.

Here, $U_{k}^{\left(j,n\right)}(x,y),\;n=1,2$, the terms of this representation, are solutions of the Lamé equations for a homogeneous half-plane satisfying the boundary conditions

(4) $$t_{}^{\left(j,1\right)}(0,y)=\mu_{j}\;X_{}^{\left(j,1\right)}(y),\;\;\;\;\;\;t_{}^{\left(j,2\right)}(h_{j},y)=\mu_{j}\;X_{}^{\left(j,2\right)}(y)$$

We represent the displacement vector $U_{}^{\left(j,1\right)}(x,y)$ in the form of a Fourier integral through the transformants of the stress vector $X_{}^{\left(j,1\right)}(y)$

(5) $$U_{}^{\left(j,1\right)}(x,y)=\frac{1}{2\pi }\int_{\Gamma }P_{}^{\left(j,1\right)}(x,\alpha )\cdot {\tilde{X}}_{}^{\left(j,1\right)}(\alpha )e^{-i\alpha y}d\alpha$$

The contour Γ is determined by the principle of limiting absorption: it bypasses the positive poles of the integrand from below, negative from above, and coincides with the real axis in the rest of the part.

Matrix components $P^{\left(j,1\right)}$ are given in Appendix A and depend on relative frequencies $\theta_{j1}=\frac{\omega }{V_{pj}};\;\theta_{j2}=\frac{\omega }{V_{Sj}}$, where $V_{pj},V_{sj}$ are the wave speeds for corresponding medium.

To take into account the viscoelastic properties of the medium, the loss tangents are introduced for longitudinal and shear waves $\mathrm{tg}\gamma_{p}^{\left(j\right)},\;\mathrm{tg}\gamma_{s}^{\left(j\right)}$, equal to ratio of imaginary parts of relative frequencies squares to real ones tgγp(j)=Im(θj12)Re(θj12),  tgγs(j)=Im(θj22)Re(θj22). In this case, contour Γ equals the real axis completely.

Similar to formula (5), the displacements for the half-plane $x\leq h_{j}$ through functions ${\tilde{X}}_{}^{\left(j,2\right)}(\alpha )$ are determined, where the elements $P_{nm}^{\left(j,2\right)}(x,\alpha )$ satisfy the relations

(6) $$P_{nm}^{\left(j,2\right)}(x,\alpha )={\left(-1\right)}^{\delta_{nm}}P_{nm}^{\left(j,1\right)}(h_{j}-x,\alpha ),\;n,m=1,2$$

$\delta_{nm}$ is the Kronecker symbol.

Determining the stress state of the layer as the sum of the corresponding solutions for two half-planes, we obtain

(7) $${\left\{\sigma_{x},\tau_{xy}\right\}}_{}^{\left(j,1\right)}{}^{T}(x,y)=\frac{1}{2\pi }\int_{\Gamma }W_{}^{\left(j,1\right)}(x,\alpha )\cdot {\tilde{X}}_{}^{\left(j,1\right)}(\alpha )e^{-i\alpha y}d\alpha$$

where $W^{\left(j,1\right)}(x,\alpha )$ are given in Appendix B.

For the second group of terms, we find

(8) $$W_{nm}^{\left(j,2\right)}(x,\alpha )={\left(-1\right)}^{\delta_{nm}+1}W_{nm}^{\left(j,1\right)}(h_{j}-x,\alpha )$$

The functions $U_{k}^{{}^{\left(j,3\right)}}$ determine displacements in a homogeneous plane with the parameters of the layer under consideration from the action of a concentrated source of oscillations $p(r_{0})=\left\{p_{1}(x_{0},y_{0}),p_{2}(x_{0},y_{0})\right\}$ in the form of a set of cylindrical waves and correspond to the components of the matrix $U^{*}(r_{0},r)$

(9) $$U_{k}^{\left(j,3\right)}(x_{0},y_{0},x,y)=\sum_{l=1}^{2}U_{kl}^{\left(j,3\right)}(x_{0},y_{0},x,y)\;\;p_{l}(x_{0},y_{0});\;\;k=1,2$$

Using the decomposition formulas, they can be written in the Fourier transform form

(10) $$\begin{array}{l} {\tilde{U}}_{kl}^{\left(j,3\right)}(x_{0},y_{0},x,\alpha )=F_{y}[U_{kl}^{\left(j,3\right)}]=\int_{-\infty }^{+\infty }U_{kl}^{\left(j,3\right)}(x_{0},y_{0},x,y)\exp(i\alpha y)\;dx\\ U_{kl}^{\left(j,3\right)}(x_{0},y_{0},x,y)=F_{\alpha }^{-1}[{\tilde{U}}_{kl}^{\left(j,3\right)}]=\frac{1}{2\pi }\int_{\Gamma }^{}{\tilde{U}}_{kl}^{\left(j,3\right)}(x_{0},y_{0},x,\alpha )\exp(-i\alpha y)\;dx\\ k,l=1,2 \end{array}$$

The form of functions ${\tilde{U}}_{kl}^{\left(j,3\right)}\left(x_{0},y_{0},x,\alpha \right)$ is given in Appendix A.

Following the same logics for fundamental solutions for stresses, we get:

(11) $$T_{kn}^{\left(j,3\right)}(x_{0},y_{0},x,y)=\sum_{l=1}^{2}T_{lkn}^{\left(j,3\right)}(x_{0},y_{0},x,y)\;\;p_{l}(x_{0},y_{0});\;\;k,n=1,2$$

$T_{lkn}^{\left(j,3\right)}\left(x_{0},y_{0},x,y\right)$ in Fourier transformation form are given in Appendix B.

The introduced Fourier transforms of the stress functions ${\tilde{X}}_{}^{\left(j,k\right)}(\alpha )$ of representations (2), (7) are unknown and must be determined from the conditions for joining the dissimilar components of the layered half-plane with each other. Satisfying the equalities of the components of the vectors of displacements and stresses when passing through the interfaces between the media in the Fourier transforms, we obtain a system of linear algebraic equations 4N+2  with unknowns

(12) $$A(\alpha )\cdot \tilde{X}(\alpha )=B(\alpha )$$

where $\tilde{X}(\alpha )$ is the general vector of unknown stresses for a multilayer structure.

In the frictionless case and under coupling conditions, the view of matrix A(α) will change. This leads to change in dispersion properties of construction and difference in properties for wave’s refraction and transition through partition boundary which is also presented in [31,32]. In particular, for road related constructions, the effects of smooth contact lead to decreasing in structural properties and physically to redistribution of vibration energy to higher frequency area especially in side zones around loading area. Such effect can be used for methods of road coatings quality inspection.

The fundamental solutions obtained in this way have the important property of the absence of stresses on the day surface $x=x_{N}$. This excludes from participation in representations (1) unknown displacements on this part of the boundary of the region D.

Letting the point $r_{0}$ be further to the boundary of the area occupied by the foundation, we obtain a system of boundary integral equations for the vectors $u_{}^{\left(0\right)}$ and τ

$$\begin{array}{c} \frac{1}{2}u_{}^{\left(0\right)}(r_{0})-\int_{\gamma }^{}T^{*}(r_{0},r)\cdot n(r)\cdot u_{}^{\left(0\right)}(r)ds-\int_{\Lambda }^{}U^{*}(r_{0},r)\cdot \tau (r)ds=\\ =\int_{\Omega }^{}{U^{*}(r_{0},r)|}_{x=0}\cdot q(y)dy,\;r_{0}\in \gamma , \end{array}$$

(13) $$\begin{array}{c} \frac{1}{2}u_{}^{\left(0\right)}(r_{0})+\int_{\gamma }^{}T^{**}(r_{0},r)\cdot n(r)\cdot u_{}^{\left(0\right)}(r)ds-\int_{\Lambda }^{}U^{**}(r_{0},r)\cdot \tau (r)ds=\\ =\int_{\Omega }^{}{U^{**}(r_{0},r)|}_{x=0}\cdot q(y)dy,\;r_{0}\in \Lambda . \end{array}$$

The system of Equations (13) is solved by the method of linear or constant boundary elements with subsequent spline approximation of the obtained distributions of unknown functions γ and Λ. The centers of boundary elements and docking nodes are chosen as collocation points.

Using the solution to system (13) and subsequent numerical differentiation, the authors consider the concentration of dynamic stresses at the boundary of the foundation depending on its depth and the ratio of the elastic parameters of the foundation layers.

**Figure 2:**
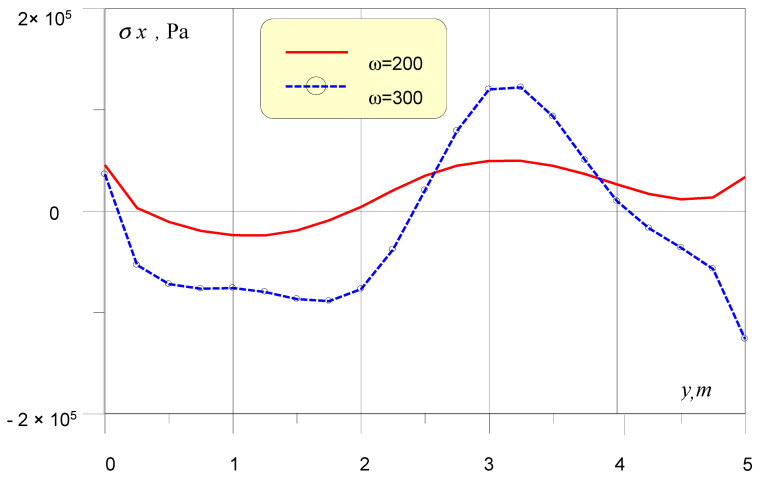
Normal stresses on lower edge of foundation.

As a computational example, Figure 2 represents $\sigma_{x}$ normal stresses distribution on lower edge of buried by 0.4 m in triple layered foundation of 0.5 m height. The soil foundation structure is supposed to be normal with increasing of wave speeds values along the layers thickness. The dynamic load is created by surface point sources, moved from foundation by 2.5 m and with the level of 100 kN. The plots represent cases of source oscillations with frequencies ω = 200 and 300 rad/s. The figure shows that stresses distribution can have areas of equal sign, which leads to bending and rotation of the foundation.

## 3. Experimental Methods

### 3.1. Experimental Technique

**Figure 3:**
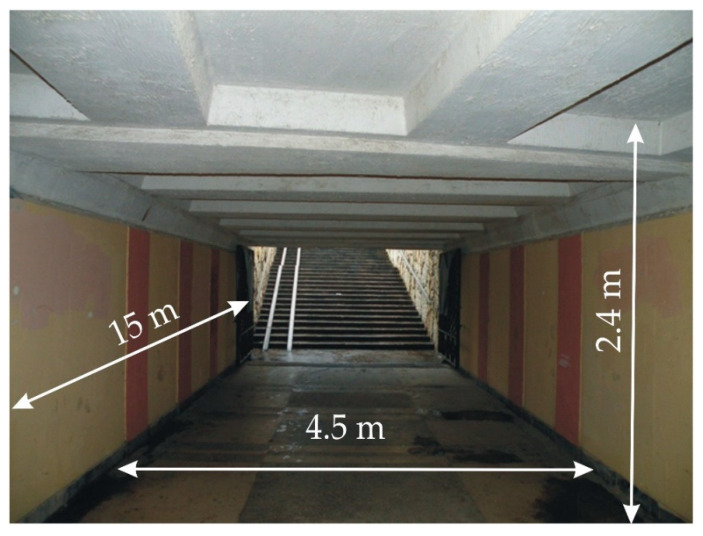
Underground pedestrian crossing.

Experimental studies were carried out at an underground pedestrian crossing located in the city center of Rostov-on-Don, Russia. The total volume of the structure is up to 1200 m^3^, the depth of the footing is up to 3.8 m, and the depth of occurrence under the carriageway above the upper beams is 0.5 m. The view of the pedestrian crossing is shown in Figure 3.

**Figure 4:**
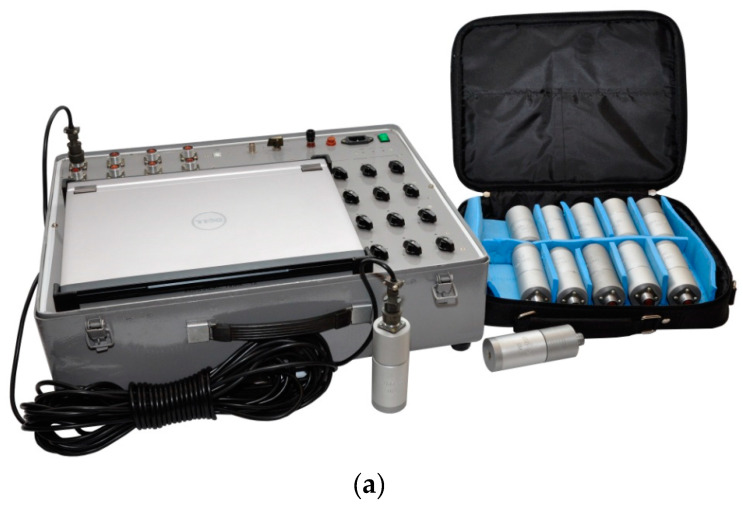

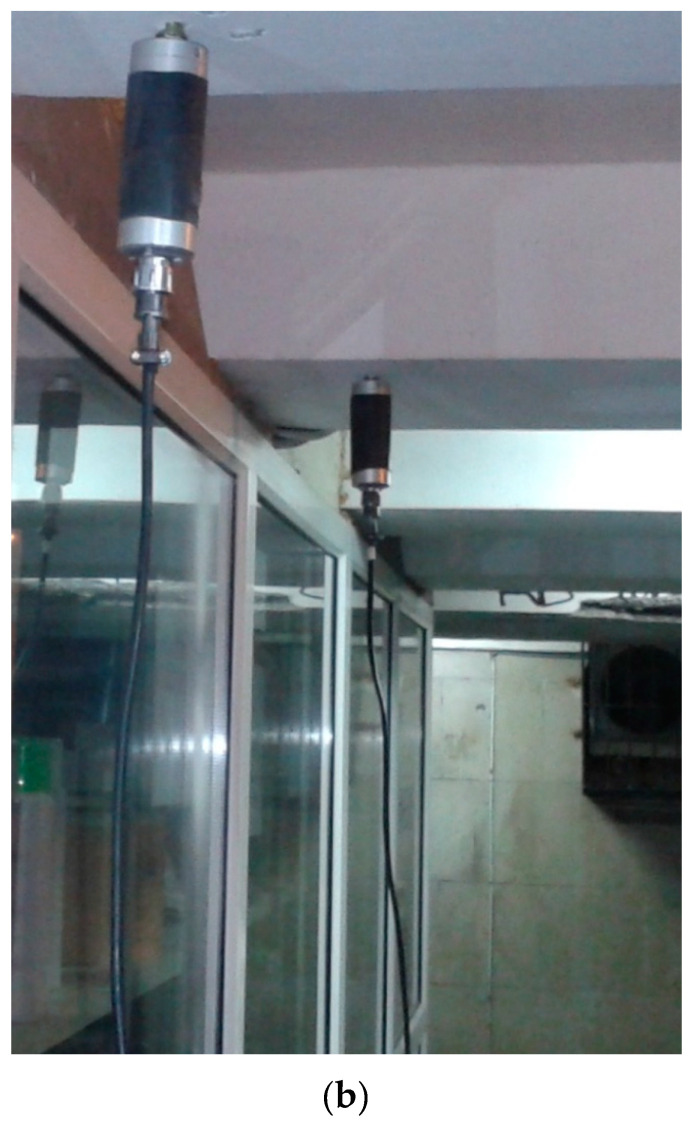
Vibration measuring device (**a**); and scheme of the sensors installation (**b**).

Vibration measuring 12-channel device VK-12 (Figure 4) is developed and implemented on the basis of the E14–440 module manufactured by the company “L-Card” the level of noise (micro seismic vibrations) and transmission of signals in digital form for further processing in a computer (Table 1).

During experimental studies, the accelerometers sensors were fixed in the center of the ceilings using anchor bolts. This type of fastening provides the best match between the sensor and the overlap.

During experimental studies, the level of vibration velocities from the following sources was investigated:

(1) passage of a KamAZ vehicle weighing 20.5 tons at a speed of 30 km/h;
(2) passage of a KamAZ vehicle weighing 20.5 tons at a speed of 50 km/h;
(3) impact on the floor.

Programs for signal processing are based on the transformation of data arrays, and the construction of amplitude–frequency characteristics (AFC) on the basis of the obtained AVX by applying the fast Fourier transform. For a more correct result, for each series of measurements, a correlation analysis was carried out, followed by averaging and smoothing of the results.

Amplitude–frequency characteristics obtained from ABX using fast Fourier transform reflect the integral values of the amplitudes for each of the frequencies, in a given interval of the original signal.

It should also be noted that, when choosing the method of vibrations excitation for evaluating the mechanical properties of surface layers and the structural state of the shallow laying object, it is possible to use effectively such diagnostic installations as falling weight of large mass HWD.

### 3.2. Results Processing Technique

In accordance with GOST R 52892–2007, the vibration velocities were obtained using the integral Fourier transform to the recorded amplitude–time characteristics of the vibration accelerations. Namely, considering the properties of the Fourier transform, as well as that $A(t)=\dot{V}(t)$, the following dependence is valid

(14) $$\bar{V}(\omega )=\frac{\bar{A}(\omega )}{i\omega }$$

Here, ω is the circular frequency of oscillations, *i* is the imaginary unit, *A*(*t*) and *V*(*t*) are the accelerations and velocities of oscillations, $\bar{V}(\omega ),\;\bar{A}(\omega )$ are the Fourier images of the velocities and accelerations of oscillations, respectively, and

(15) $$\bar{V}(\omega )=\int_{-\infty }^{+\infty }V(t)\exp(-i\omega t)dt$$

(16) $$\bar{A}(\omega )=\int_{-\infty }^{+\infty }A(t)\exp(-i\omega t)dt$$

Thus, having registered vibration accelerations, the transition to vibration velocities was carried out by the formula

(17) $$V(t)=\frac{1}{2\pi }\int_{-\infty }^{+\infty }\frac{\bar{A}(\omega )}{i\omega }\exp(i\omega t)d\omega$$

For the experimental determination of the frequency of natural bending vibrations of the beam, we used the results of recording the vibration accelerations under impact.

The shock impact corresponds to a Gaussian pulse [31]

(18) $$p(t)=P\exp\left(-a^{2}t^{2}\right)$$

Here, *P* is the peak value of the shock load and 2/a is the duration of the pulse. The spectrum of a Gaussian pulse is determined by the formula

(19) $$S(\omega )=P\int_{-\infty }^{+\infty }\exp\left(-a^{2}t^{2}\right)\exp\left(-i\omega t\right)dt=\frac{P\sqrt{\pi }}{a}\exp\left(-{\left(\frac{\omega }{2a}\right)}^{2}\right)$$

where ω=2πf is the circular vibration frequency and *f* is the linear vibration frequency.

With a short exposure time, the spectrum of a Gaussian pulse is close to a constant over a wide frequency range.

Under external influence on the structure, the spectrum of the recorded response depends on the spectrum of external influence and the spectral characteristics of the structure. This dependence is expressed by the formula

(20) $$S_{0}=S_{B}S_{K}$$

where *S*_0_ is the response spectrum, *S_B_* is the spectrum of external influences, and *S_K_* is the structure’s own spectrum. Then, the spectrum of the structure is determined by the formula

(21) $$S_{K}=\frac{S_{0}}{S_{B}}$$

Thus, it follows from formula (21) that under impact action the response spectrum will differ very little from the structure’s own spectrum.

Since the direct registration of the dynamic response of the structural elements of the transition to an arbitrary dynamic impact is difficult due to the large flow of pedestrians and vehicles, the calculated response spectra from the reference load can be used to assess the impact of an arbitrary non-stationary load on the structural elements. In this study, the impact was taken as a reference effect when a KamAZ vehicle with a mass of 20.5 t passed at speeds of 30 and 50 km/h.

To determine the dynamic response to an arbitrary dynamic action, it is necessary to do the following:

Perform test registration of the response to the dynamic impact under investigation in an arbitrary place (for example, on a road with low traffic volume).

Register the response to the reference action in the same place.

Get the design response by the formula

(22) $$V(t)=\frac{1}{2\pi }\int_{-\infty }^{+\infty }\frac{{\bar{V}}_{NS}(\omega )}{{\bar{V}}_{ES}(\omega )}{\bar{V}}_{E}(\omega )\exp(i\omega t)d\omega$$

where ${\bar{V}}_{ES}(\omega )$ is the spectrum of response rates to the reference impact, ${\bar{V}}_{NS}(\omega )$ is the spectrum of response rates to the investigated non-stationary impact, and ${\bar{V}}_{E}(\omega )$ is the spectrum of response rates to the reference impact recorded on the structural elements of the pedestrian crossing.

## 4. Results and Discussion

As an example of the application of the proposed calculation method, let us consider the problem of determining the dynamic stresses on the inner boundary of a buried object caused by loads on the day surface of a multilayer medium. The problem is relevant when analyzing the strength and performance of underground structures. One of the approaches to its solution is to establish links between the maximum (peak) voltage values and the maximum values of vibration velocities recorded by sensors at measurement points. When considering the vibrations of beams (slabs) as elements of ground structures, usually GOST R 52892–2007 uses a ratio of the form

(23) $$\sigma \leq \sqrt{E\rho }\sqrt{\frac{3G_{tot}}{G}}k_{n}^{}\nu_{\max}$$

where, in accordance with GOST R 52892–2007, E is dynamic modulus of elasticity of the beam; ρ is the density of the beam material; $G_{tot}$ is the total weight of the beam, including dead weight G and surface load; $k_{n}$ is modal coefficient corresponding to the fundamental mode of vibration, having a value in the range from 1 to 1.33; and $v_{\max}$ is the maximum value of the vibration velocity along the entire length of the beam

However, the presence of a layered medium, into which a buried object is immersed, does not allow applying this formula directly to underground structures and requires taking into account the processes of wave propagation in inhomogeneous media.

Note also that, when waves propagate in homogeneous media, the relationship between the maximum values of stresses and velocities is preserved. Thus, for the simplest case of wave propagation from a surface lumped oscillating ω source of shear in the z direction of the axis, the displacements have the form

(24) $$u_{z}(x,y)=-\frac{i}{2}H_{0}^{\left(1\right)}(\theta \sqrt{x^{2}+y^{2}})$$

where $\theta =\frac{\omega }{\sqrt{\mu /\rho }}$ is the reduced vibration frequency, $H_{0}^{\left(1\right)}$ is the Hankel function of the first kind, and μ is the shear modulus of the material. Hence, it is easy to show that, for all frequencies with distance $r=\sqrt{x^{2}+y^{2}}>>1$ from the source, $\frac{\sigma_{zr}}{v_{\max}}\leq \sqrt{\mu \rho }$.

Thus, in the case of an object immersed in a layered medium, it can be established that

(25) $$\sigma_{y}\leq \sqrt{E\rho }\cdot C(E_{i},\rho_{i},h_{i})\cdot \nu_{\max}$$

The dimensionless characteristic function $C(E_{i},\rho_{i},h_{i})$ depends on the material properties of the components of the layered medium and the thicknesses of the layers $h_{i}$, i=1,…,N, primarily located above the object.

**Figure 5:**
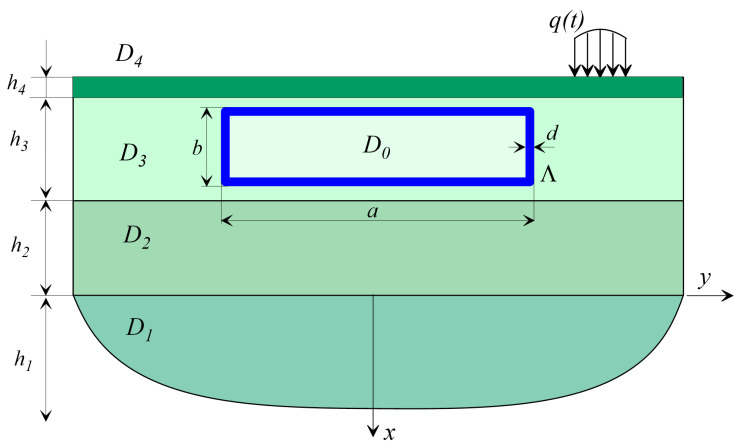
Model for calculating the dynamic response of an object *D*_0_ in a multilayer medium *D*_1_, *D*_2_, *D*_3_ and *D*_4_.

**Figure 6:**
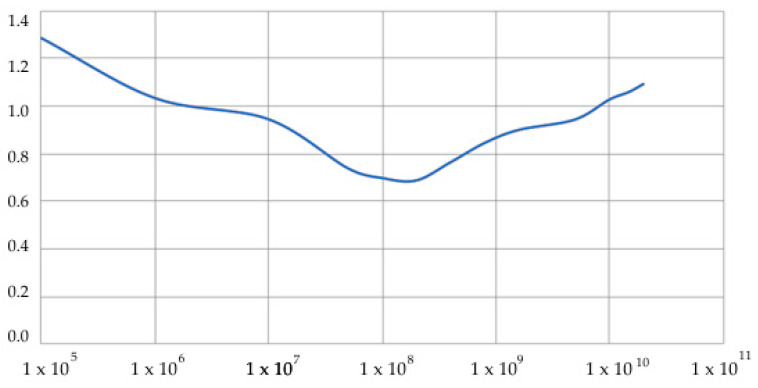
Dependence $C(E_{i},\rho_{i},h_{i})$ on the modulus of elasticity of the immersion layer *E*_3_, Pa.

**Figure 7:**
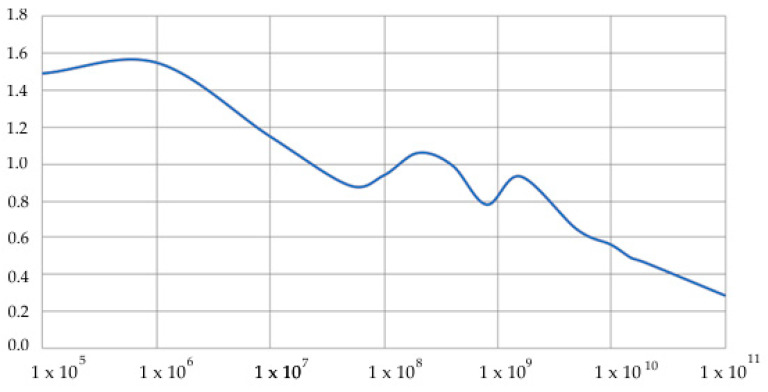
Dependence $C(E_{i},\rho_{i},h_{i})$ on the modulus of elasticity of the immersion layer *E*_3_, Pa, $h_{4}=1$.

As examples of calculations (Figure 5), a problem is considered in which an object is located at a depth in Layer 3 and experiences dynamic effects *q*(*t*). Figure 6 and Figure 7 show the dependences $C(E_{i},\rho_{i},h_{i})$ on the parameters of the problem.

### 4.1. The Underground Object Is Buried in the D_3_ Layer and the D_4_ Layer Is Missing

Consider the problem of the dynamics of an underground object located according to the scheme in Figure 5, when the *D*_4_ layer is absent. The properties of the media (parameters with index 0 refer to an underground facility) are as follows: *E*_0_ = 2 e^4^ MPa, *E*_1_ = 100 MPa, *E*_2_ = 80 MPa, $\rho_{0}$ = 2400 kg/m^3^, $\rho_{1}$ = 2000 kg/m^3^, and $\rho_{2}$ = 1900 kg/m^3^. Layer thicknesses are $h_{1}=\infty$, $h_{2}=4$ m, and $h_{3}=4$ m. Since there is no *D*_4_ layer, *h*_4_ = 0. Object dimensions are *a* = 6 m and *b* = 2.5 m. The wall thickness is 0.3 m. Depth of the object is $h_{0}=1.2$ m.

Figure 6 shows the results of calculating the characteristic function $C(E_{i},\rho_{i},h_{i})$ depending on the elastic modulus of the layer in which the object is immersed. A wide range of elastic moduli is considered, from *E* = 1 × 10^5^ Pa (that is, silty soils) to *E* = 1 × 10^10^ Pa (granite and harder). Figure 5 shows that soft layers and very hard layers increase the characteristic function $C(E_{i},\rho_{i},h_{i})$, and, in the zone of materials corresponding to sandy loams and loams, the characteristic function is close to 1. This makes it possible to effectively use dependence (25) in a wide range of experimental data.

### 4.2. Underground Object Buried in D_3_ Layer and D_4_ Layer Available

Medium properties are as follows: *E*_0_ = 2 e^4^ MPa, *E*_1_ = 100 MPa, *E*_2_ = 80 MPa, *E*_3_ = 200 MPa, $\rho_{0}$ = 2400 kg/m^3^, $\rho_{1}$ = 2000 kg/m^3^, $\rho_{2}$ = 1900 kg/m^3^, and $\rho_{3}$ = 2000 kg/m^3^. Layer thicknesses are $h_{1}=\infty$, $h_{2}=4$ m, $h_{3}=4$ m, and $h_{4}=1$ m. Object dimensions are *a* = 6 m and *b* = 2.5 m. Wall thickness is 0.3 m. Depth of the object is $h_{0}=0.2$ m.

Figure 7 shows the dependence of the characteristic function on the modulus of elasticity of the surface layer *E*_4_; the layer height is conventionally assumed to be 1 m.

Figure 7 shows that, in soft layers, when *E*_4_ of the surface layer *D*_4_ is less than the elastic modulus of the underlying layer *E*_3_, that is, *E*_4_ << *E*_3_, the characteristic function increases significantly, which is due to resonance phenomena in a layered medium. The upper layer resonates and makes a significant nonlinear contribution to the general picture of the dynamics of a layered medium. If the modulus of elasticity of the near-surface layer *E*_4_ is close to the modulus of the underlying layer *E*_3_, the characteristic function is close to 1. In the case when the rigidity of the surface layer *E*_4_ is greater than the underlying *E*_3_, that is, *E*_4_ >> *E*_3_, the harder layer dampens all resonances, being a kind of low-frequency filter in the system, and the characteristic function drops to 0.3.

**Figure 8:**
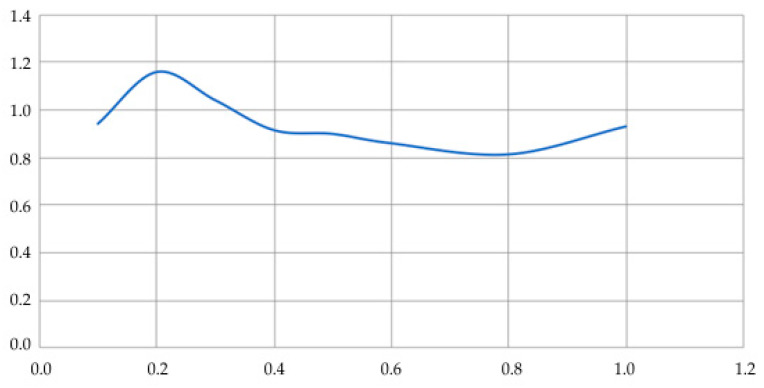
Dependence $C(E_{i},\rho_{i},h_{i})$ on thickness *h*_4_ of the surface layer *E*_4_ at $E_{4}=1600$ MPa (asphalt concrete).

Figure 8 shows the dependence of the characteristic function on the thickness of the surface layer. Asphalt concrete with a modulus of elasticity *E*_4_ = 1600 MPa was taken as a layer material.

Figure 8 shows that for the accepted value of the modulus of elasticity *E*_4_ = 1600 MPa in a wide range of layer thickness *D*_4_; the characteristic function is in the range from 0.8 to 1.2, which makes it possible to effectively apply dependence (25) in practical measurements of the stress–strain state.

**Figure 9:**
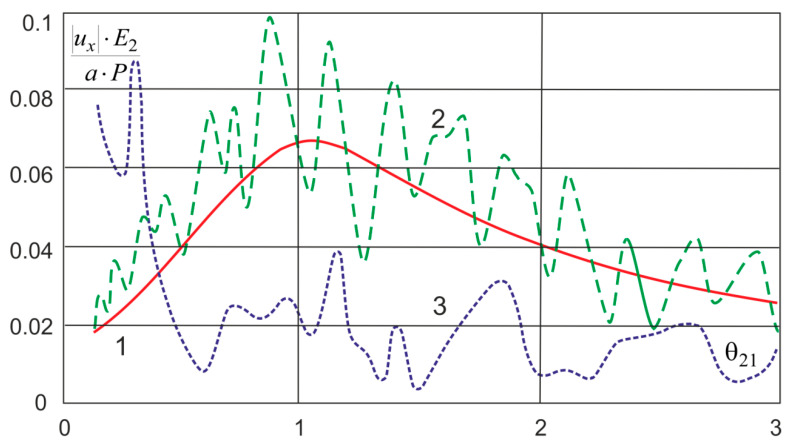
Dependence of the amplitude–frequency characteristics of the layered medium: 1, half-space; 2, softer near-surface layer *E*_4_ < *E*_3_; 3, harder near-surface layer *E*_4_ > *E*_3_.

Figure 9 shows the dependences of the amplitude–frequency characteristics of the layered medium. Here, 1 is a half-space; 2 is a softer near-surface layer *E*_4_ < *E*_3_; and 3 is a harder near-surface layer *E*_4_ > *E*_3_. *P* is the load, *a* is the coordinate of the point of deepening of the object, and *E*_2_ is the elastic modulus of the half-space. The dependence is plotted in dimensionless quantities.

Figure 9 shows that the softer near-surface layer *E*_4_ < *E*_3_ resonates and, due to multiple reflections from the layer boundaries, gives a change in the vibration amplitude. This effect differs from the results obtained by Pryakhina et al. [33], since, in our case, a massive object differs from a solid inclusion. A tougher layer works differently. Only in the low-frequency region there are still resonance frequencies corresponding to bending vibrations; in the rest of the range, resonances are damped by the upper hard layer. The results obtained are in good agreement with those of Zhou et al. [24], who obtained a solution for a cylindrical region in a layered medium.

**Figure 10:**
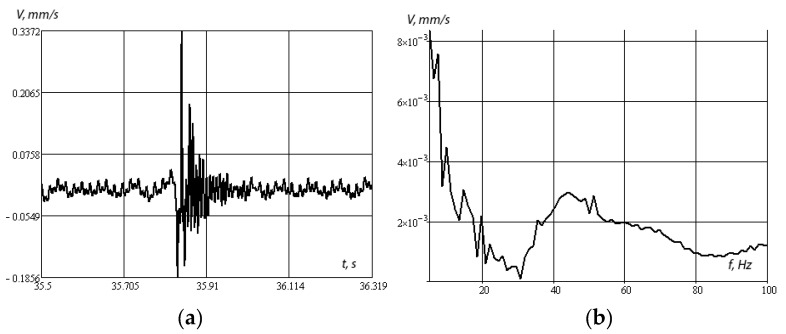
Amplitude–time (**a**) and amplitude–frequency (**b**) characteristics of vibration velocities, impact load.

Figure 10a,b shows the amplitude–time and amplitude–frequency dependences under shock loading.

Figure 10b shows that, under shock loading, the most energetic resonances lie in the low-frequency region and in the region of about 43 Hz, and then the frequency response fades. Note that the presence of low-frequency maxima of the amplitude–frequency characteristic is explained by the presence of a massive object immersed in a layered medium as a whole. In the case of significant rigidity of the underlying half-space, such maxima can be significant. In the limiting case of a rigidly clamped layer, the presence of isolated low-frequency resonances for a surface object was considered in the works of I.I. Vorovich [34,35].

**Figure 11:**
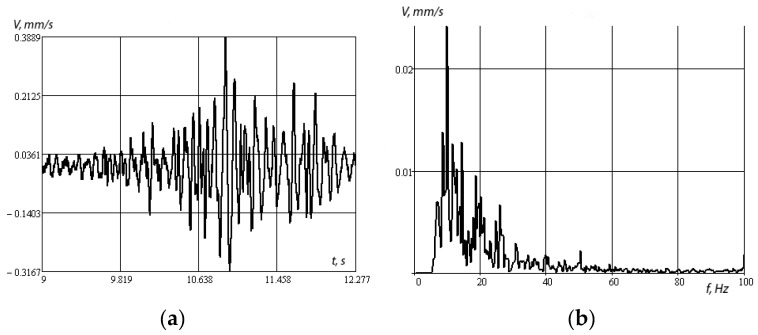
Amplitude–time (**a**) and amplitude–frequency (**b**) characteristics of vibration velocities, passage of a Kamaz car at a speed of 50 km/h.

Figure 11a,b shows the amplitude–time and amplitude–frequency dependences when a KamAZ car is driven at a speed of 50 km/h.

Figure 11 shows that the energy resonances lie in the low-frequency region. Resonance frequencies at 10, 17, and 19 Hz are due to the frequency of external influences when driving a car. Accordingly, the response of the structure changes its vibration spectrum.

**Figure 12:**
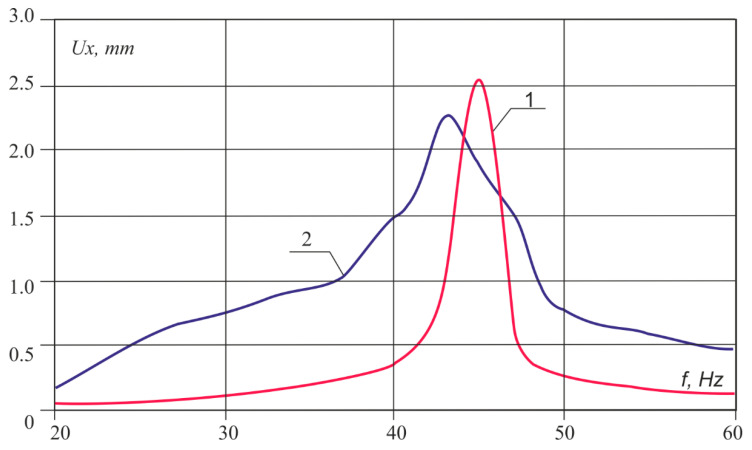
Response spectra of underpass overlap: 1, theoretical; 2, experimental.

Figure 12 shows an example of calculating the overlap spectrum using the amplitude–frequency characteristic of the vibration velocities of the overlap under impact and the Gaussian pulse spectrum. The figure clearly shows a peak around 42 Hz. It should be noted that, when passing vehicles on the amplitude–frequency characteristics, resonance peaks were not observed at frequencies above 20 Hz. It follows from this that the frequency range of the impact of vehicles lies below the natural frequencies of bending vibrations of floors.

## 5. Conclusions

The article discusses the problem and proposes a method for monitoring underground structures of arbitrary shape, lying in layered media, under various external dynamic influences. An analytical solution to the problem of the dynamics of layered media with buried objects of arbitrary shape is obtained. On the example of an underground passage, a practical example of the estimation of resonance frequencies and characteristic function for various characteristics and geometry of layers, is considered.

The instrumental analysis of the stress–strain state is complicated by the fact that at the characteristic function, which depends on the properties of the medium and has a nonlinear character. The frequency ranges in which the characteristic function is close to 1 are identified and recommendations are given for processing sensor signals for buried objects.

It is shown that the softer near-surface layer *E*_4_ < *E*_3_ resonates and, due to multiple reflections from the layer boundaries, gives a change in the vibration amplitude. A tougher layer works differently. Only in the low-frequency region there are still resonance frequencies corresponding to bending vibrations; in the rest of the range, resonances are damped by the upper hard layer.

As part of the research methodology development, it is proposed to use it in solving of inverse structural mechanics problems for reconstruction of buried object properties by measuring and analyzing vibration responses on a medium surface and an internal object geometry, as well as in the development of linear consolidation theory methods for layered water-saturated soils.

## Figures and Tables

**Table 1:** Main characteristics of the vibration measuring device.

1	Dynamic range of input levels	50 db
2	Bit depth analog-to-digital Converter	12 bit
3	Power consumption of the IV-1 device	<250 mA
4	Complex measurement error	<5%
5	Operating temperature range	0 °C … +55 °C
6	Conversion rate	>2500 mW/m^−1^ s^2^

## Appendix A

Components of displacements matrix:

(A1) $$P_{11}^{\left(j,1\right)}(x,\alpha )=\frac{\sigma_{j1}}{\Delta_{R}}\left\{-\zeta_{j}^{2}e_{j1}+2\alpha^{2}e_{j2}\right\}$$

(A2) $$P_{12}^{\left(j,1\right)}(x,\alpha )=\frac{i\alpha }{\Delta_{R}}\left\{-2\sigma_{j1}\sigma_{j2}e_{j1}+\zeta_{j}^{2}e_{j2}\right\}$$

(A3) $$P_{21}^{\left(j,1\right)}(x,\alpha )=\frac{i\alpha }{\Delta_{R}}\left\{-\zeta_{j}^{2}e_{j1}+2\sigma_{j1}\sigma_{j2}e_{j2}\right\}$$

(A4) $$P_{22}^{\left(j,1\right)}(x,\alpha )=\frac{\sigma_{j2}}{\Delta_{R}}\left\{2\alpha^{2}e_{j1}-\zeta_{j}^{2}e_{j2}\right\}$$

(A5) $$\Delta_{R}=-4\alpha^{2}\sigma_{j1}\sigma_{j2}+\varsigma_{j}^{4},\;e_{jk}=e^{-\sigma_{jk}^{}x}$$

(A6) $$\zeta_{j}^{2}=\alpha^{2}+\sigma_{j2}^{2}\;,\;\sigma_{jk}^{2}=\alpha^{2}-\theta_{jk}^{2}\;\;$$

(A7) $${\tilde{U}}_{11}^{\left(j,3\right)}(x_{0},y_{0},x,\alpha )=A_{j}\left[-\sigma_{j1}E_{j1}^{}+\alpha^{2}E_{j2}^{}/\sigma_{j2}\right]$$

(A8) $${\tilde{U}}_{12}^{\left(j,3\right)}(x_{0},y_{0},x,\alpha )={\tilde{U}}_{21}^{\left(j,3\right)}(x_{0},y_{0},x,\alpha )=i\alpha A_{j}\left[E_{j1}^{}-E_{j2}^{}\right]\mathrm{sign}(x-x_{0})$$

(A9) $${\tilde{U}}_{22}^{\left(j,3\right)}(x_{0},y_{0},x,\alpha )=A_{j}\left[-\sigma_{j2}E_{j2}^{}+\alpha^{2}E_{j1}^{}/\sigma_{j1}\right]$$

(A10) $$\;E_{jk}^{}=\exp(-\sigma_{jk}\left|x_{0}-x\right|),\;\;k=1,2;\;A_{j}=\frac{\exp(i\alpha y_{0})}{2\theta_{j2}^{2}}$$

## Appendix B

Components of stresses matrix:

(A11) $$W_{11}^{\left(j,1\right)}(x,\alpha )=\frac{1}{\Delta_{R}}\left\{\zeta_{j}^{4}e_{j1}-4\sigma_{j1}\sigma_{j2}\alpha^{2}e_{j2}\right\}$$

(A12) $$W_{12}^{\left(j,1\right)}(x,\alpha )=\frac{2i\alpha \sigma_{j2}\zeta_{j}^{2}}{\Delta_{R}}\left\{e_{j1}-e_{j2}\right\},$$

(A13) $$W_{21}^{\left(j,1\right)}(x,\alpha )=\frac{2i\alpha \sigma_{j1}\zeta_{j}^{2}}{\Delta_{R}}\left\{e_{j1}-e_{j2}\right\}$$

(A14) $$W_{22}^{\left(j,1\right)}(x,\alpha )=\frac{1}{\Delta_{R}}\left\{-4\sigma_{j1}\sigma_{j2}\alpha^{2}e_{j1}+\zeta_{j}^{4}e_{j2}\right\}$$

(A15) $${\tilde{T}}_{111}^{\left(j,3\right)}(x_{0},y_{0},x,\alpha )=A_{j}\left[\varsigma_{j}^{2}E_{j1}^{}-2\alpha^{2}E_{j2}^{}\right]\mathrm{sign}(x-x_{0})$$

(A16) $${\tilde{T}}_{211}^{\left(j,3\right)}(x_{0},y_{0},x,\alpha )=\frac{i\alpha A_{j}}{\sigma_{j1}}\left[\varsigma_{j}^{2}E_{j1}^{}-2\sigma_{j1}\sigma_{j2}E_{j2}^{}\right]$$

(A17) $$({\tilde{T}}_{112}^{\left(j,3\right)}(x_{0},y_{0},x,\alpha )=\frac{i\alpha A_{j}}{\sigma_{j2}}\left[2\sigma_{j1}\sigma_{j2}E_{j1}^{}-\varsigma_{j}^{2}E_{j2}^{}\right]$$

(A18) $${\tilde{T}}_{212}^{\left(j,3\right)}(x_{0},y_{0},x,\alpha )=A_{j}\left[-2\alpha^{2}E_{j1}^{}+\varsigma_{j}^{2}E_{j2}^{}\right]\mathrm{sign}(x-x_{0})$$

(A19) $${\tilde{T}}_{122}^{\left(j,3\right)}(x_{0},y_{0},x,\alpha )=A_{j}\left[-\eta_{j}^{2}E_{j1}^{}+2\alpha^{2}E_{j2}^{}\right]\mathrm{sign}(x-x_{0})$$

(A20) $${\tilde{T}}_{222}^{\left(j,3\right)}(x_{0},y_{0},x,\alpha )=\frac{i\alpha A_{j}}{\sigma_{j1}}\left[-\varsigma_{j}^{2}E_{j1}^{}+\sigma_{j2}\left(2-\frac{\theta_{j2}^{2}}{\theta_{j1}^{2}}\left(1-\frac{\sigma_{j2}}{\sigma_{j1}}\right)\right)E_{j2}^{}\right]$$

(A21) $$\eta_{j}^{2}=2\alpha^{2}+\frac{\lambda_{j}}{\mu_{j}}\theta_{j1}^{2}$$
